# Potential role for microRNA-16 (miR-16) and microRNA-93 (miR-93) in diagnosis and prediction of disease progression in mycosis fungoides in Egyptian patients

**DOI:** 10.1371/journal.pone.0224305

**Published:** 2019-10-24

**Authors:** Iman Mamdouh Talaat, Rania ElSaied Abdelmaksoud, Maha Guimei, Naglaa Fathi Agamia, Ahmed Nugud, Ahmed Taher El-Serafi

**Affiliations:** 1 Clinical Sciences Department, College of Medicine, University of Sharjah, Sharjah, UAE; 2 Research Institute for Medical & Health Sciences, University of Sharjah, Sharjah, UAE; 3 Department of Pathology, Faculty of Medicine, Alexandria University, Alexandria, Egypt; 4 Department of Dermatology, Andrology and Venereology, Faculty of Medicine, Alexandria University, Alexandria, Egypt; 5 Pediatric Resident, Aljalila Children Hospital, Dubai, UAE; 6 Medical Biochemistry and Molecular Biology Department, Faculty of Medicine, Suez Canal University, Ismailia, Egypt; Wayne State University, UNITED STATES

## Abstract

Mycosis Fungoides (MF) is the most common type of cutaneous T-cell lymphomas. Early stage patients are treated with topical therapies and have normal life expectancy whereas patients with advanced disease encounter frequent relapses and have a five-year survival rate that does not exceed 15%. The aim of the present study was to characterize the expression of microRNA-16 (miR-16) and microRNA-93 (miR-93) in early and advanced cases of MF in relation to the clinicopathological parameters. Ten skin biopsies of early and advanced MF were investigated for the expression of miR-16 and miR-93 using RT-PCR. Immunohistochemical expression of apoptosis markers (BCL-2 and Survivin) were also investigated in the studied cases compared to normal skin and eczema biopsies. In the present study, BCL-2 and Survivin showed strong positive expression on neoplastic lymphocytes in all cases of MF regardless of their stage. We have also shown that miR-16 was significantly upregulated in advanced cases of MF compared to cases with early disease (p-value was less than 0.05). However, expression of miR-16 did not show any statistically significant correlation with age, gender, or expression of apoptotic markers. On the other hand, the expression of miR-93 showed significant downregulation in all lymphoma cases irrespective of their stage, compared to normal and eczema cases. Our results suggest that upregulation of miR-16 could be used to predict an aggressive course of the disease. We also suggest that miR-93 downregulation could serve as possible tool for establishing early diagnosis in early challenging cases. Our findings also provide consistent evidence that the anti-apoptotic molecules may play an important role in the pathogenesis of this type of cutaneous lymphomas and promote the idea that their inhibition could be an interesting novel therapeutic strategy in the treatment of MF.

## Introduction

Cutaneous T-cell lymphomas (CTCLs) are rare types of non-Hodgkin lymphomas (NHLs) of the skin. The most common form of which is mycosis fungoides (MF). It accounts for around 55–60% of the new cases of CTCL diagnosed every year whereas Sézary syndrome (SS), its leukemic variant, accounts for 5% of the cases [1]. In MF, malignant T-cells are described singly or in clusters in the epidermis; a phenomenon known as “epidermotropism”. They form “Pautrier microabscesses” which are collections of malignant T-cells adherent to the processes of Langerhans cells. With progression of the disease, epidermotropism is gradually lost together with an increase in the number of malignant, and a decrease in non-malignant, infiltrating T-cells [2].

Patients with early stages of the disease (stage IA, IB) can remain undiagnosed for years as they present with flat erythematous skin patches or plaques that resemble both clinically and histologically other inflammatory diseases such as dermatitis or psoriasis, which renders the pathological diagnosis of MF in these cases quite challenging. Whereas in the later stages, the disease assumes tumorous forms, has a more aggressive clinical course and a markedly reduced 5- year survival [3].

Staging and treatment stratification of CTCL follows the 2005 classification of the European organization for research and treatment of cancer (EORTC) and the World Health organization (WHO) [4]. This classification depends on TNMB (Tumor, node, metastasis, blood) as the main prognostic parameter that forms the basis for treatment planning [5]. Patients with patch/plaque disease are usually staged IA-IB and are known to have “limited-stage” MF. Their overall survival is measured in decades and, in patients with stage IA, it is comparable to normal age-matched population. On the other hand, patients with advanced stage disease, and those who show significant leukemic involvement (B2) are considered to have “advanced-stage” MF. In these patients, the disease is considered incurable and the median survival of patients ranges between 1–5 years [6]. In spite of the great advances achieved in treatment of MF and SS, available topical and systemic therapies have resulted in decreased tumor burden and improved quality of life, but have offered limited effects on patient survival [7]. Therefore, the search for novel molecular markers that could enable early diagnosis of the disease as well as markers that could present possible therapeutic targets is still needed in order to improve the outcome of patients with advanced disease.

To date, the molecular pathogenesis of CTCL remains poorly understood. Several studies have suggested that dysfunctional regulation of the apoptotic pathways is strongly involved in the pathogenesis and progression of CTCL [8–11]. Inhibiting apoptosis by upregulating BCL2 transcription, increases BCL2 activity and results in progressive tumor growth [12, 13]. Currently, the most effective treatments for MF/SS, such as phototherapy [14], photopheresis [15] and even systemic therapies act by enhancing apoptosis of malignant T-cells. Therefore, targeting apoptosis and apoptosis related genes and proteins seems like a highly promising treatment strategy for these patients. The role of apoptosis in CTCL has been further highlighted in a recent study that showed that concurrent inhibition of BCL2 and HDAC (Histone Deacetylase) offered synergy in the treatment of CTCL and achieved a more effective and personalized treatment for these patients [7].

Other anti-apoptotic genes and proteins have been less studied in CTCL like Survivin, which is a member of the inhibitor of apoptosis (IAP) family of proteins [16]. This marker is not expressed in normal and differentiated cells but abundant expression could be shown in various tumors, including tumors of the lung, breast, pancreas, malignant melanomas as well as certain types of non-Hodgkin lymphomas (NHLs) [16, 17]. It promotes cell proliferation and inhibits apoptosis by interacting with effector caspases in the cytoplasm [17]. It is known to contribute to tumor growth and maintenance hence has been suggested as an attractive potential therapeutic target. Few recent studies have also suggested a significant increase in Survivin gene and protein expression in MF and SS compared to normal CD4+T cells [18].

Epigenetic regulation of gene expression has been lately recognized as an important key player in tumorigenesis. This can be—at least partially—mediated by noncoding RNA such as microRNA (miRNA) that exert their effects by post transcriptional regulation of gene expression. miRNA expression was found to be deregulated in malignant tumors resulting in disruption of several biological processes including cell proliferation, differentiation, invasiveness, as well as apoptosis. miRNA may contribute to cancer initiation and progression, thus exhibiting an oncogenic role or they may show tumor suppressor properties, depending on their target genes [19].

Recently, there has been increasing evidence to suggest that miRNAs play an important role in the pathogenesis of lymphoid malignancies including CTCLs [20, 21]. Several studies showed differential expression of miRNAs between early and advanced cases of MF. These include miR-93, miR-15b, miR-17, miR-21, miR-25, miR-31, miR-92a-1, miR-106b, miR-107, miR-191, miR-425, and miR-769-5p. Thus indicating a role for these types of miRNA in disease progression [22]. Among those, miR-93 has shown to be overexpressed in several malignancies including ALK + anaplastic large cell lymphoma (ALCL) [23], gastric as well as hepatocellular carcinomas [24]. Yet, its exact role in MF has not been fully elucidated.

Another miRNA that has also been closely linked to human malignancies is miR-16 [25]. miR-16 is known to be frequently deleted and/or down-regulated in many types of cancers, such as chronic lymphocytic leukemia (CLL) [26] prostate cancer and lung cancer [27]. miR-16 modulates the cell cycle, inhibits cell proliferation, promotes cell apoptosis and suppress tumorigenicity both *in vitro* and *in vivo* [28]. In CLL, another type of NHL, miR-16-1 expression was also found to be inversely correlated with BCL2 expression and that it negatively regulated BCL2 at a post-transcriptional level [29].

In view of the important role of miRNA in tumorigenesis in general, and in CTCL in specific, our aim in the present study was to characterize the expression of miR-16 and miR-93 in early and advanced cases of MF in relation to apoptosis and to the different clinicopathological parameters.

## Materials and methods

### Ethics statement

The research was approved by the Research Ethics Committee of the Faculty of Medicine, Alexandria University (Alexandria, Egypt). Biopsies from the patients with MF and eczema were collected after informed consent according to the Helsinki declaration.

### Tumor samples

This study was retrospectively conducted on formalin-fixed paraffin-embedded (FFPE) tissue obtained from the archives of the Pathology Department, Alexandria University, Egypt. The material consisted of 12 cases of eczema, 10 cases of MF as well as 5 control normal skin biopsies. The histological diagnoses were established using hematoxylin and eosin (H&E) stain and were confirmed using immunohistochemistry (CD3 and CD4). The diagnoses were revised by 2 independent pathologists (IT and MG). The clinical data were retrieved from the Dermatology Department, Alexandria University, Egypt. According to TNMB staging system (5), patients with MF were further subdivided into 7 ‘limited stage’ cases and 3 cases of ‘advanced MF’(S1 Table).

### BCL2 and Survivin immunohistochemistry

Immunohistochemical staining was performed using FFPE serial sections (5 μm thick) from eczema and MF cases. Tissue sections were deparaffinized in xylene and rehydrated in descending concentrations of alcohols, then placed in phosphate-buffered saline (PBS) for 5 minutes. After rehydration, the sections were boiled in citrate buffer (pH 6) for antigen retrieval then were incubated with the primary antibodies. All antibodies were purchased from Abcam, Cambridge, UK. Rabbit monoclonal were used; anti-BCL2 antibody (cat no. ab32124), anti-Survivin antibody (cat no. ab134170) were used at a concentration of 1:250 and 1:100 respectively. Slides were incubated overnight in a moist chamber, washed in tris-buffered saline (TBS), then incubated with rabbit anti-mouse secondary antibody for 30 minutes at room temperature. The sections were developed with anti-rabbit HRP/DAB detection kit (cat no. ab64261). Sections were washed in running tap water and lightly counterstained with hematoxylin, followed by dehydration and coverslip mounting. Positive and negative controls were included in each run.

### miRNA extraction

miRNA was extracted from paraffin embedded tissue using miRNeasy FFPE Kit (Qiagen, Germany), according to the manufacturer’s protocol. Briefly, 4 to 5 sections of 10 μm thickness were incubated in xylene for dissolving paraffin, followed by incubation with a special buffer to reverse the formaldehyde modification of nucleic acids then with proteinase-K and DNase. miRNA was isolated using a special column and eluted in RNase-free water. The RNA yield was checked on a NanoDrop- 1000 spectrophotometer (Thermo Scientific, USA). All samples displayed absorbance ratios of 260/280 above 1.6 and were included in the qPCR analysis.

### miRNA expression

The extracted miRNA was reversely transcribed using miScript II RT Kit (Qiagen, Germany), with miScript HiFlex Buffer. The expression level was studied using Real Time PCR with miSCript Sybr Green Kit (Qiagen, Germany). The sequence of the primers is listed in “Table 1”.

**Table 1:** miR-16 and miR-93 primers sequence.

Primer	Sequence	Reference
**Mir-16-5p_forward**	5’-TAGCAGCACGTAAATATTGGCG-3’	https://www.nature.com/articles/srep30824#t2 https://www.ncbi.nlm.nih.gov/pmc/articles/PMC6348261/ https://www.hindawi.com/journals/bmri/2019/1759697/
**Mir-93-3p_ forward**	5’-ACACTCCAGCTGGGCAAAGTGCTGTTCGTGC-3’	https://www.nature.com/articles/s41598-019-42309-4 https://www.ncbi.nlm.nih.gov/pmc/articles/PMC5464472/ https://www.wjgnet.com/1007-9327/full/v17/i42/4711-T1.htm https://www.ncbi.nlm.nih.gov/pubmed/26035737
**Universal miRNA reverse**	5’-GTGCAGGGTCCGAGGT-3’	https://www.nature.com/articles/ncb2910#supplementary- https://www.ncbi.nlm.nih.gov/pmc/articles/PMC6348261/ https://www.ncbi.nlm.nih.gov/pmc/articles/PMC2225395/
**U6_forward**	ATTGGAACGATACAGAGAAGATT	https://www.nature.com/articles/s41598-017-01027-5
**U6_reverse**	GGAACGCTTCACGAATTTG	https://www.nature.com/articles/s41598-017-01027-5

### Statistics analysis

GraphPad Prism version 7.00 for Windows (GraphPad Software, La Jolla California USA) was used for statistical analysis. miRNA expression level was calculated using the delta Ct method and corrected to the level of expression of U6. Significant level between the three studied groups (healthy, eczema and MF) was studied by using nonparametric rank-based test (Kruskal-Wallis). p-value was considered significant when less than 0.05. For multinomial regression, SPSS Inc. Released 2007. SPSS for Windows, Version 16.0. Chicago, SPSS Inc was used.

### Results

**Fig 1:**
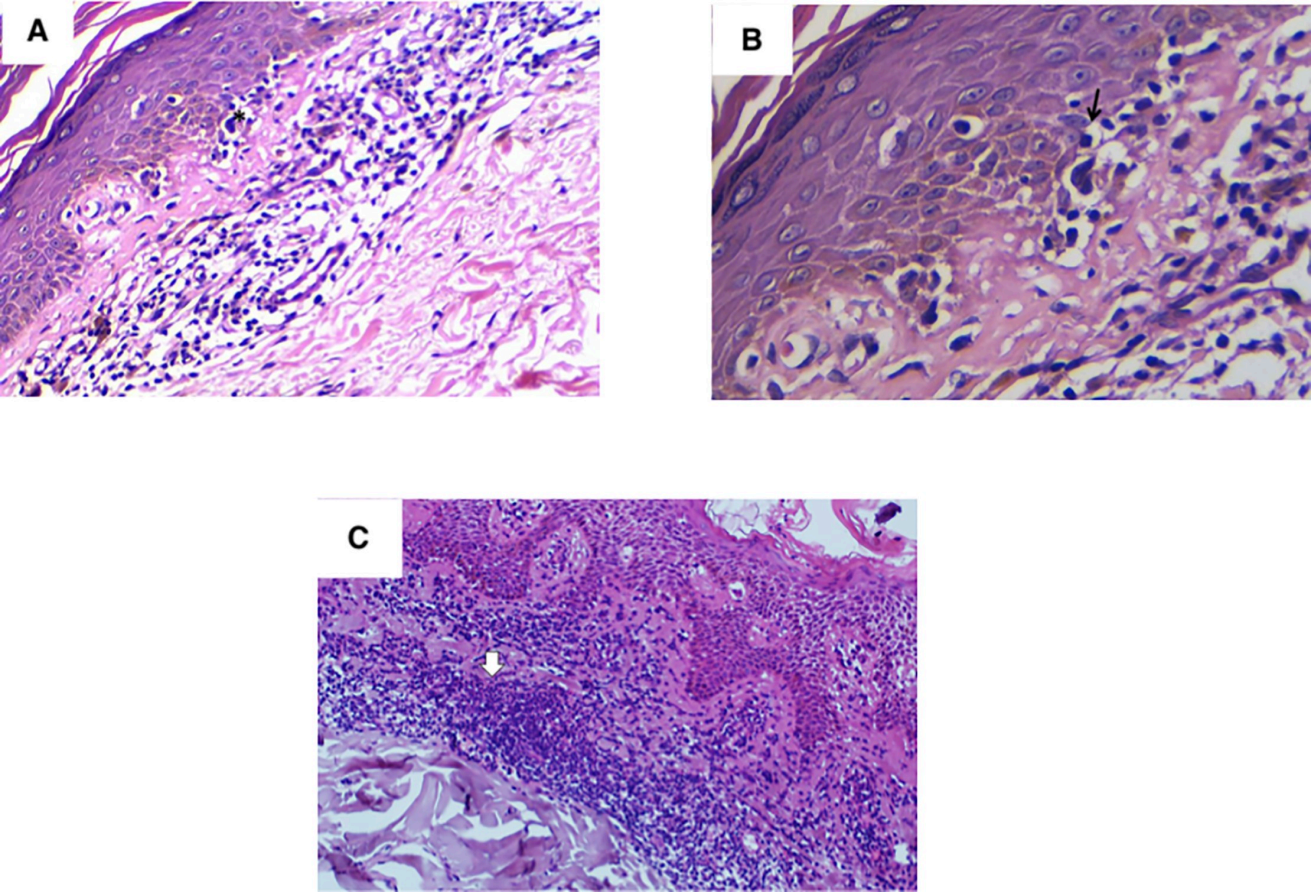
Different H&E representative sections of Mycosis fungoides cases. (A) A case of mycosis fungoides exhibiting lymphocyte epidermotropism where malignant lymphocytes are arranged along the basal layer of the epidermis surrounded by a clear halo (*). (B) A higher power view of the same patient showing “Pautrier microabscess” (arrow) (H&E, x200). (C) Another case of mycosis fungoides featuring hyperchromatic lymphocytes and a band-like dermal lymphoid infiltrate (arrow) amid background of dermal fibroplasia (H&E, x200).

The present study was conducted on 10 cases of MF, 12 cases of eczema and 5 normal skin biopsies used as control (S1 Table). According to the TNMB staging system, MF cases were divided into 2 groups; the first comprised 7 cases of ‘limited MF’ (patch/ plaque stage); 3 cases of which were stage (IA) and 4 cases had stage (IB), the second group consisted of 3 cases with “advanced MF” (tumor stage); one case stage (IIA), and 2 cases staged as (IIB) “Fig 1”.

### Immunohistochemical expression of BCL2 and Survivin

**Fig 2:**
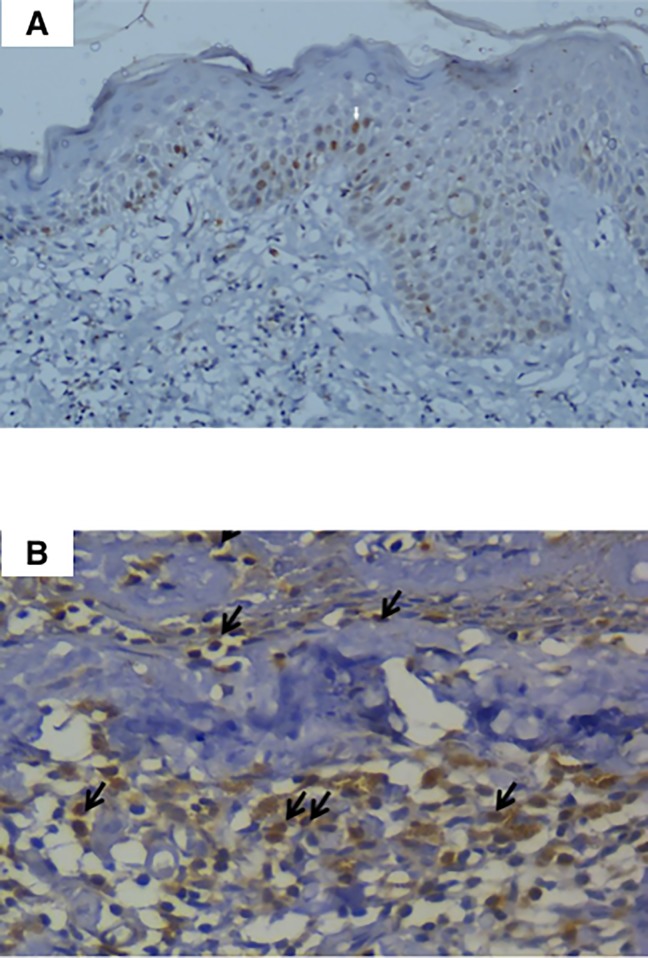
BCL2 expression in normal and MF cases. (A) A section showing normal skin revealing scattered nuclear positivity (arrow) in the keratinocytes (Immunoperoxidase, x200). (B) A case of MF showing strong BCL2 nuclear expression in neoplastic T-cells in both the dermis and the epidermis (arrows) (Immunoperoxidase, x200).

BCL2 immunohistochemical expression was noted in keratinocytes in all cases of eczema (100%), as well as in all control skin biopsies (100%), “Fig 2A”. In cases of “limited stage” MF, immunohistochemical expression of BCL2 was noted in the nuclear membrane of the neoplastic lymphocytes “Fig 2B” with the exception of one case. Similarly, among the 3 studied cases of “advanced MF”, only one case (30%) showed no expression of BCL2 whereas the other cases were strongly positive for BCL2. In the present study, expression of BCL2 was consistently positive in lymphocytes of most MF cases (both limited and advanced disease stages). However, its expression did not show any statistically significant correlation with age, gender or stage of the disease.

**Fig 3:**
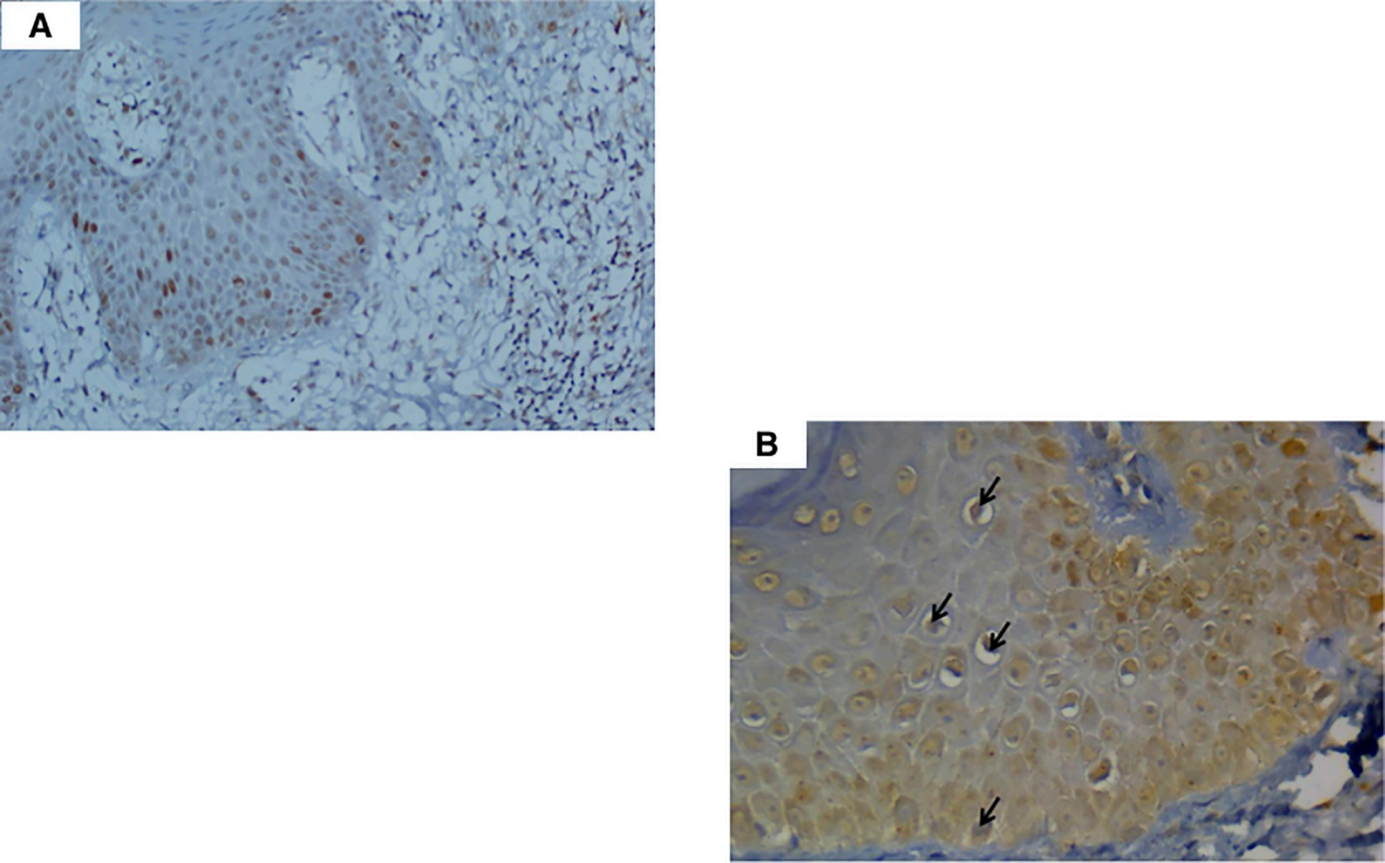
Survivin expression in normal and MF cases. (A) A case of eczema exhibiting nuclear positivity (arrow) in the keratinocytes (Immunoperoxidase, x200). (B) A case of MF showing positive nuclear expression of Survivin in the epidermal malignant lymphocytes (arrows) (Immunoperoxidase, x400).

Similar to BCL2, the immunohistochemical expression of the anti-apoptotic protein, Survivin was consistently positive in keratinocytes of all eczema “Fig 3A” and control cases. As for MF cases, Survivin showed strong nuclear expression in the lymphocytes “Fig 3B” in all except one case of MF that belonged to the advanced stage group (stage IIB). Survivin expression in the studied cases did not show any statistically significant correlation with age, gender or stage of the disease.

### miR-16 and miR-93 expression in limited versus advanced MF

**Fig 4:**
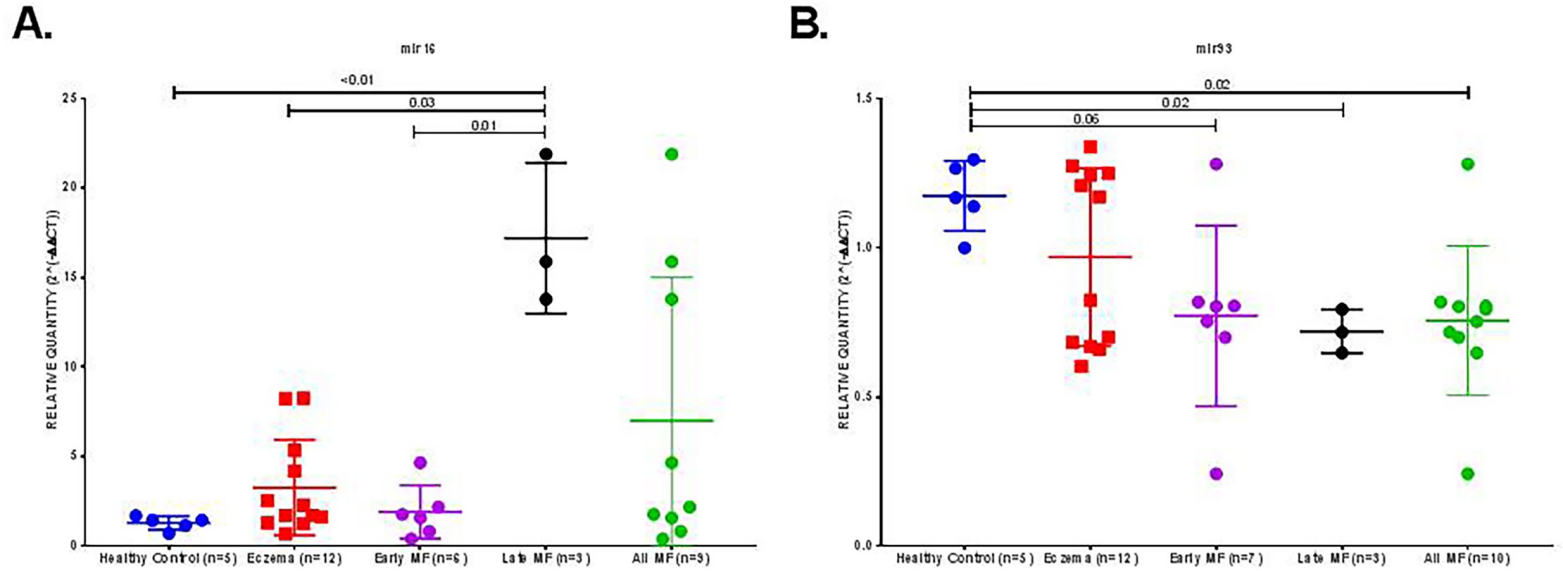
Relative gene expression of miR-16 and miR-93 measured by qRT PCR in skin samples from healthy controls (n = 5), eczema patients (n = 12), early MF (n = 7) and Late MF (n = 3). Non parametric Kruskal-Wallis test was used to test if one of the groups has significant difference in miR-16 and miR-93 expression compared to others. (A) Relative Quantification of miR-16 in all the studied groups showing that late MF express higher level of miR-16 compared to early MF (p = 0.01), eczema (p = 0.03) and healthy controls (p<0,01). (B) Relative Quantification of miR-93 in all the studied groups showing that in healthy controls, miR-93 was more expressed compared to late MF (p = 0.02) and all MF if grouped together (p = 0.02).

Next, we compared miR-16 expression in limited MF versus advanced stage MF. miR-16 expression was found to be significantly higher in advanced MF cases compared to cases of limited MF (p = 0.01) and cases of eczema (p = 0.03) and normal controls (P<0.01) “Fig 4A”. This result suggests that miR-16 may have a possible role in predicting an aggressive course of the disease and determining disease prognosis.

Expression of miR-16 did not show any statistically significant correlation with age, gender, or stage. It did not also show any correlation with the studied apoptotic markers (BCL2 and Survivin) that were equally expressed in all cases of MF regardless of their stage.

Interestingly, the expression of miR-93 showed significant downregulation in all MF cases, compared to normal (p = 0.02) “Fig 4B”. There was no statistical difference between advanced and limited MF in term of miR-93 expression, indicating that it is a possible MF specific marker.

Non parametric Kruskal-Wallis test was used to test if one of the groups had significant difference in miR-16 and miR-93 expression compared to others. It was significant for miR-16 (P = 0.03) but not significant in miR-93 (P = 0.12).

Using miR-16 and miR-93 expression as independent predictors whether a patient is healthy (control) or suffering from MF or eczema was assessed by multinomial logistic regression. Using miR-16 and miR-93 expression showed a significant effect on the logistic regression model compared to “Intercept Only” model (p = 0.04) “Tables 2 and 3”.

**Table 2:** Nominal regression, model fitting information.

Model	Model fitting criteria	Likelihood ratio tests		
	-2 Log likelihood	Chi-square	df	Sig.
**Intercept only Final**	54.139			
	38.754	15.384	4	0.004

**Table 3:** Nominal regression, likelihood ratio tests.

Effect	Model fitting criteria	Likelihood ratio tests		
	-2 Log likelihood of reduced model	Chi-square	df	Sig.
**Intercept**	41.379	2.625	2	0.269
**Mir16**	45.842	7.087	2	0.029
**Mir93**	47.038	8.284	2	0.016

If we used MF group expression as a reference to predict whether the patient is healthy or presenting with eczema, a unit change in miR-93 expression, is expected to change the logit of outcome “being healthy” 7.8 times given that miR-16 is held constant. These results indicate that Using miR-16 and miR-93 expression can serve as an independent predicator for patient’s diagnosis with MF and can differentiate between the early versus advanced stage of MF “Table 4”.

**Table 4:** Logit of outcome.

Diagnosis ^a^		B	Standard error	Wald	df	Sig.	Exp (B)	95% confidence interval for Exp (B)	
								Lower bound	Upper bound
**Eczema**	Intercept	-1.570	1.689	0.864	1	0.353			
	Mir16	-0.131	0.110	1.429	1	0.232	0.877	0.708	1.087
	Mir93	2.890	1.875	2.376	1	0.123	18.001	0.456	710.464
**Healthy**	Intercept	-4.879	3.824	1.628	1	0.202			
	Mir16	-1.962	1.563	1.576	1	0.209	0.141	0.007	3.007
	Mir93	7.835	3.696	4.493	1	0.034	2.528E3	1.804	3541755.935

^**a**^ The reference category is MF

## Discussion

Resistance to apoptosis remains a major obstacle for the success of cancer treatment. In CTCL, most of the currently used therapeutic modalities like phototherapy and photopheresis are directed towards enhancing apoptosis of tumor cells [14, 15]. Nevertheless, none of these modalities, including the newly introduced agents like retinoids, histone deacetylase (HDAC) inhibitors or pralatrexate have efficiently achieved improvement of survival particularly when it comes to “advanced stage” CTCL patients who still to date suffer a dismal outcome [30]. This, in turn, highlights the urgent need to identify those cases that are more prone to disease progression and to develop novel effective therapeutic modalities that could limit tumorigenesis in these patients.

In the present study, we investigated the expression of two of the most widely described anti-apoptotic proteins (BCL2 and Survivin) in MF. They exert their anti-apoptotic effect by two different mechanistic pathways; BCL2 regulates the intrinsic apoptosis pathway by decreasing the release of cytochrome c into the cytoplasm and thereby controlling cytoplasmic caspase activation [13] while, Survivin directly inhibits cytoplasmic caspases [17]. We found that BCL2 was strongly expressed on the neoplastic T-cells in almost all cases of CTCLs, regardless of their stage. On the other hand, its expression in eczema and control cases was only limited to basal keratinocytes. However, the limited number of cases in our study did not allow an accurate comparison between its expression in the early and late stages of the disease.

Our findings are consistent with the previous studies that showed that both BCL2 and p53 were expressed in peripheral T-cell lymphomas (PTCL), including CTCL, and constituted an independent prognostic factor that correlated significantly with the progression of the disease [12]. A recent next generation sequencing study have also identified “Dysregulation of apoptosis” as one of 3 key genetic derangements occurring in CTCL [8]. Studies on leukemic CTCL using newly developed FISH probes have also identified four common genetic alterations; amplification of STAT3 and STAT5B and deletions of P53 and CTLA4. All of which are linked to inhibition of apoptosis by upregulating the anti-apoptotic protein BCL2 [12]. Results from our study and these previous studies emphasize the oncogenic role of BCL2 in CTCL and highlight the fact that it could present a potential therapeutic target. In a recent interesting study, the *in-vitro* effect of the BCL2 inhibitor Venetoclax (ABT-199) was investigated on survival of the malignant cells isolated from the peripheral blood of CTCL patients [7]. The majority of CTCL patient samples showed strong sensitivity to Venetoclax, and the levels of BCL2 expression were negatively correlated to 50% inhibitory concentration values. Hence, they suggested that the addition of Venetoclax to CTCL current treatment with a histone deacetylase (HDAC) inhibitor, could improve the therapeutic response. Venetoclax is the only and the most recently approved BCL2 inhibitor by the US Food and Drug Administration (FDA). It has been approved for treatment of relapsed or refractory CLL. In the recent years, it has been also used in clinical trials for follicular lymphoma, diffuse large B-cell lymphoma, acute myeloid leukemia, multiple myeloma, Waldenstrom macroglobulinemia, and NHL except CTCL [31].

In the present study, we also demonstrated that Survivin expression was noted on the neoplastic T-cells of almost all patients with MF regardless of their stage. Alternatively, Survivin expression was only limited to keratinocytes in eczema patients. To the best of our knowledge, very few studies have investigated Survivin expression in MF. In accordance with our findings, a study reported a significant upregulation in the expression of Survivin gene and protein using RT-PCR and ELISA, respectively, in cases of MF compared to normal controls [18]. Another more recent study has found that Survivin was expressed in SS patients' Sézary cells but not in healthy donors' CD4+ T cells, and that Avicin-D-induced apoptosis in these cells was partially dependent on downregulation of STAT-3 and the anti- apoptotic proteins (BCL2 and Survivin) [32]. Our findings, in agreement with the previous studies, provide consistent evidence that the anti-apoptotic molecules may play an important role in the pathogenesis of CTCL and promote the idea that their inhibition could represent an interesting novel therapeutic strategy in the treatment of CTCL. However, the exact molecular mechanisms that affect their expression still needs to be further explored.

In the recent years, the discovery of the epigenetic regulation of gene expression and the altered miRNA expression in cancer has revolutionized our understanding of the physiological and pathological processes in the human body. It has also made clear that interfering with miRNA expression, whether alone or in combination with other modalities, could be used as a novel therapeutic target for cancer therapy. Despite the important role played by miRNAs in the pathogenesis of several cancers, very few studies have elucidated their exact role in CTCL.

In the present study we have investigated the expression of miR-16 and miR-93 in MF. We have shown that miR-16 expression was significantly upregulated in ‘advanced disease’ CTCL compared to ‘limited MF’ and eczema cases. To the best of our knowledge, this finding is relatively novel. It highlights the fact that miR-16 might have an oncogenic role promoting the progression of the disease and its upregulation can be used as a possible prognostic marker that can help identify those cases that are more prone to disease progression. In contrast to our findings, most previous studies have recognized miR-16 as a tumor suppressor in various tumors including pituitary adenomas, prostatic adenocarcinomas as well as in CLL [33].

In a study on CLL cell lines, miR-16 expression was found to be downregulated in leukemic tumor cells *in–vitro* [29]. In gastric cancer cell lines, both miR-15b and miR-16 were found to be downregulated and contributed to the development of multidrug resistance (MDR) in cancer cells. Both of these studies have suggested that miR-16 exerts its tumor suppressor effect by post-transcriptionally targeting the anti-apoptotic gene BCL2 resulting in apoptosis of the tumor cells [34].

In CTCL, few studies have investigated the differential expression of miRNAs between limited and advanced MF or even MF compared to benign lesions and controls. Only one study has shown results that are in accordance with our findings. This study has investigated the complete miRome in 19 patients with MF and has found that miR-16 was among the miRNAs that were upregulated in MF in comparison to eczema cases [35]. Another study has showed that miR-15b which is also recognized as tumor suppressor in other malignancies, showed higher expression in advanced MF disease compared with MF cases with limited disease [29]. Therefore, the role of these miRNAs and their expression in MF could indicate that they are likely to be involved in disease progression.

Very limited information is available in the literature about the exact function of miR-16 in CTCL, and conflicting results have been obtained in different studies. The discrepancy in the role of the different miRNAs and in particular miR-16 in different tumors could be attributed to many factors; the nature of the studied tumor, the possibly co-existent genetic alterations as well as the different techniques used to identify its aberrant expression. A study by Tian et al [36], investigated miR-16 expression in ulcerative colitis (UC), a chronic inflammatory colonic disease characterized by accumulation of T-cells in the colonic mucosa and submucosa, and has suggested that the use of miR-16 mimics resulted in nuclear translocation and activation of NF-κ B signaling pathway with enhanced expression of IFN-γ and IL-8. NF-κB activity is known to be increased in CTCL, where it plays an important role as mediator between malignant cells and inflammatory signaling. This identified link between miR-16 and NF-κB activation could represent an alternative pathway through which miR-16 could affect tumor cell growth in MF. However further studies are still needed in order to explore how this link could affect tumorigenesis in MF.

In the present study, we demonstrated that the expression of miR-93 was significantly down-regulated in all MF cases compared to eczema and control cases. In contrast to our findings, the role of miR-93 has been investigated in several tumors and it has been mostly described as an oncomir that inhibits apoptosis and enhances cell proliferation [37]. In Non-small cell lung cancer (NSCLC), it was found that miR-93 was upregulated in comparison to non-cancerous lung tissue [38]. Its high expression in lung tumors has also been correlated with poor survival, through the downregulation of the tumor suppressor gene DAB2 [39]. Other studies have shown that mice carrying miR-93 expressing tumors had larger masses and lower survival than control mice and that the oncogenic effect was associated with angiogenesis and enhanced endothelial cell activity in these tumors [37]. Similarly, in gastric adenocarcinomas, the high expression of miR-93 detected by RT-PCR was significantly associated with a higher stage, deeper invasion, nodal metastasis and decreased overall survival [40]. In CTCL, studies have also suggested an oncogenic role for miR-93; a study by Ralfkiaer et al [23], investigated miRNAs expression in early and advanced stages of MF and has shown that miR-93 was among the miRNAs that showed higher expression in advanced cases compared to early stage disease.

Nevertheless, the literature review on the role of miR-93 in carcinogenesis has not been fully consistent and its oncogenic role could not be proved in all tumors. An interesting study on colon cancer stem cells showed that miR-93 inhibited proliferation and colony formation in colon cancer stem cells and that this suppressor effect was likely achieved via negatively targeting histone deacetylase 8 (HDAC8) [41]. HDAC inhibitors have been recently approved for treatment of MF and are known to induce cell cycle arrest and apoptosis in tumor cells [42]. This link between miR-93 and reduced histone deacetylase activity could explain the tumor suppressor role of miR-93 shown in our results. Yet this effect still needs to be further proved using a larger cohort of patients.

In summary, our results suggest that the identification of expression profiles of miRNAs could be used as potential diagnostic, prognostic as well therapeutic targets in MF. miR-93 downregulation in our MF cases suggests that it could present an additional diagnostic tool that would enable early diagnosis of limited MF cases that remain undiagnosed for years and still present a real diagnostic challenge. At the same time, miR-16 upregulation in advanced cases of MF could point to a possible prognostic role for miR-16 and could help identify those cases that are prone to disease progression. However, our data need to be further validated on a larger cohort of patients. Also, worth exploring at this point are the exact genes affected by these miRNAs and their potential targeting in treatment.

## Supporting information

S1 Table Clinicopathological characteristics of the studied groups (MF, Eczema and control). Raw data representing the age, gender, miR-16 and miR-93 expression, Bcl-2 and Survivin expression as well as the clinical and pathological stages of MF cases.
(XLSX)
